# An Evolving Clinical Phenotype Preceding the Diagnosis of Eosinophilic Granulomatosis With Polyangiitis: A Case Report

**DOI:** 10.7759/cureus.117186

**Published:** 2026-09-29

**Authors:** Mary Knowles, Pak Him Timothy Leung, Suresh Chandran

**Affiliations:** 1 Acute Medical Unit, Royal Oldham Hospital, Northern Care Alliance NHS Foundation Trust, Manchester, GBR

**Keywords:** anca-associated vasculitis, case report, delayed diagnosis, egpa, eosinophilia, eosinophilic granulomatosis with polyangiitis

## Abstract

Eosinophilic granulomatosis with polyangiitis (EGPA), formerly known as Churg-Strauss syndrome, is a rare antineutrophil cytoplasmic antibody (ANCA)-associated vasculitis characterised by eosinophilic inflammation, granulomatous disease and necrotising small- to medium-vessel vasculitis. Diagnosis is often delayed because clinical manifestations evolve gradually and mimic more common respiratory, infectious or inflammatory conditions.

We describe a 53-year-old woman with recurrent hospital admissions over a two-year period and persistent low-positive ANCA serology, during which there was initially insufficient clinical evidence of systemic vasculitis. She subsequently presented with respiratory symptoms, palpable purpura, oral ulceration and nasal crusting. Investigations demonstrated peripheral eosinophilia (9.1 × 10⁹/L) and bilateral pulmonary inflammatory infiltrates. The emergence of eosinophilia alongside pulmonary, cutaneous and upper airway involvement provided sufficient clinical evidence to support a diagnosis of EGPA. High-dose corticosteroid therapy resulted in rapid clinical improvement and normalisation of the eosinophil count.

This case highlights how EGPA may evolve over time before sufficient clinical features emerge to support the diagnosis. Recurrent non-specific presentations associated with eosinophilia and evolving multisystem involvement should prompt continued clinical reassessment, even when previous investigations have been inconclusive.

## Introduction

Eosinophilic granulomatosis with polyangiitis (EGPA) is a rare multisystem inflammatory disorder within the spectrum of antineutrophil cytoplasmic antibody (ANCA)-associated vasculitides [1,2]. It is characterised by eosinophil-rich granulomatous inflammation, necrotising small- to medium-vessel vasculitis and peripheral blood and tissue eosinophilia. Although the respiratory tract is most affected, EGPA can involve almost any organ system, including the skin, peripheral nervous system, kidneys, gastrointestinal tract and heart, resulting in a broad spectrum of clinical manifestations.

EGPA is an uncommon condition, with an estimated incidence in the United Kingdom of approximately two to four cases per million person-years and a prevalence of around 46 cases per million population [3,4]. Asthma and chronic sinonasal disease are present in the majority of patients and often precede the onset of systemic vasculitis by several years [2]. The disease is traditionally described as progressing through three phases: a prodromal phase characterised by airway disease, an eosinophilic phase with peripheral eosinophilia and eosinophilic tissue infiltration and a vasculitic phase associated with constitutional symptoms and multisystem organ involvement. However, these phases frequently overlap, and many patients do not follow the classical sequence, contributing to diagnostic uncertainty.

Diagnosis is challenging due to the lack of universally accepted criteria. Instead, diagnosis relies on assessment of the clinical presentation, laboratory investigations, imaging findings and histopathological analysis where available. Peripheral eosinophilia is one of the most consistent laboratory abnormalities, whereas ANCA are detected in only approximately 30%-40% of patients [1,5]. When present in EGPA, ANCA is most commonly directed against myeloperoxidase (MPO-ANCA), typically associated with a perinuclear ANCA (pANCA) pattern. In contrast, proteinase 3 (PR3)-ANCA is typically associated with a cytoplasmic ANCA (cANCA) pattern and is more commonly seen in granulomatosis with polyangiitis. Delayed recognition of EGPA is common because early manifestations are often non-specific and evolve gradually over months to years before the characteristic features become apparent.

We present the case of a 53-year-old woman with recurrent non-specific hospital presentations over a two-year period whose diagnosis of EGPA only became apparent following the evolution of characteristic multisystem disease. This case highlights the importance of repeated clinical reassessment in patients with persistent eosinophilia and evolving multisystem manifestations, even when earlier investigations are inconclusive.

## Case presentation

A 53-year-old woman presented to the Emergency Department following an episode of transient loss of consciousness. She reported a six-week history of cough, initially non-productive but becoming productive over the preceding two weeks, associated with central pleuritic chest pain and intermittent small-volume haemoptysis. She also described progressive nasal obstruction with crusting, painful oral ulceration and a two-week history of a painful purpuric rash involving the feet, lower limbs, upper limbs and chest.

Her past medical history included chronic obstructive pulmonary disease (COPD), chronic pancreatitis, chronic kidney disease stage 3, diverticulosis, schizophrenia and emotionally unstable personality disorder. She was an active smoker (approximately 10 cigarettes daily) and regularly used electronic cigarettes. She also consumed alcohol and had a previous history of intranasal cocaine use, which had resulted in destructive sinonasal disease. However, cocaine use had ceased prior to the index presentation.

Over the preceding two years, she had required 10 hospital admissions with recurrent community-acquired pneumonia, lower respiratory tract infections, exacerbations of COPD, cellulitis, chronic abdominal pain and acute-on-chronic pancreatitis. Intermittent purpuric skin rashes had been documented during several admissions. Persistent low-positive cANCA/PR3-ANCA serology prompted three rheumatology assessments during this period. At the earlier assessments, despite positive ANCA serology, there was insufficient clinical evidence of systemic vasculitis. Her respiratory presentations had plausible alternative explanations, including COPD and respiratory infection, while renal function was preserved, urine protein-to-creatinine ratio was normal, and there were no articular or other features of end-organ involvement. In the absence of a convincing systemic vasculitic phenotype, immunosuppressive treatment was not considered warranted and continued clinical surveillance was recommended. Retrospective review of serial laboratory investigations also demonstrated persistent eosinophilia over several months before diagnosis, with a progressive rise culminating in marked hypereosinophilia of 15.5 × 10⁹/L approximately seven weeks before the index presentation (Figure 1). On presentation, the eosinophil count remained markedly elevated at 9.1 × 10⁹/L (Figure 2).

**Figure 1:**
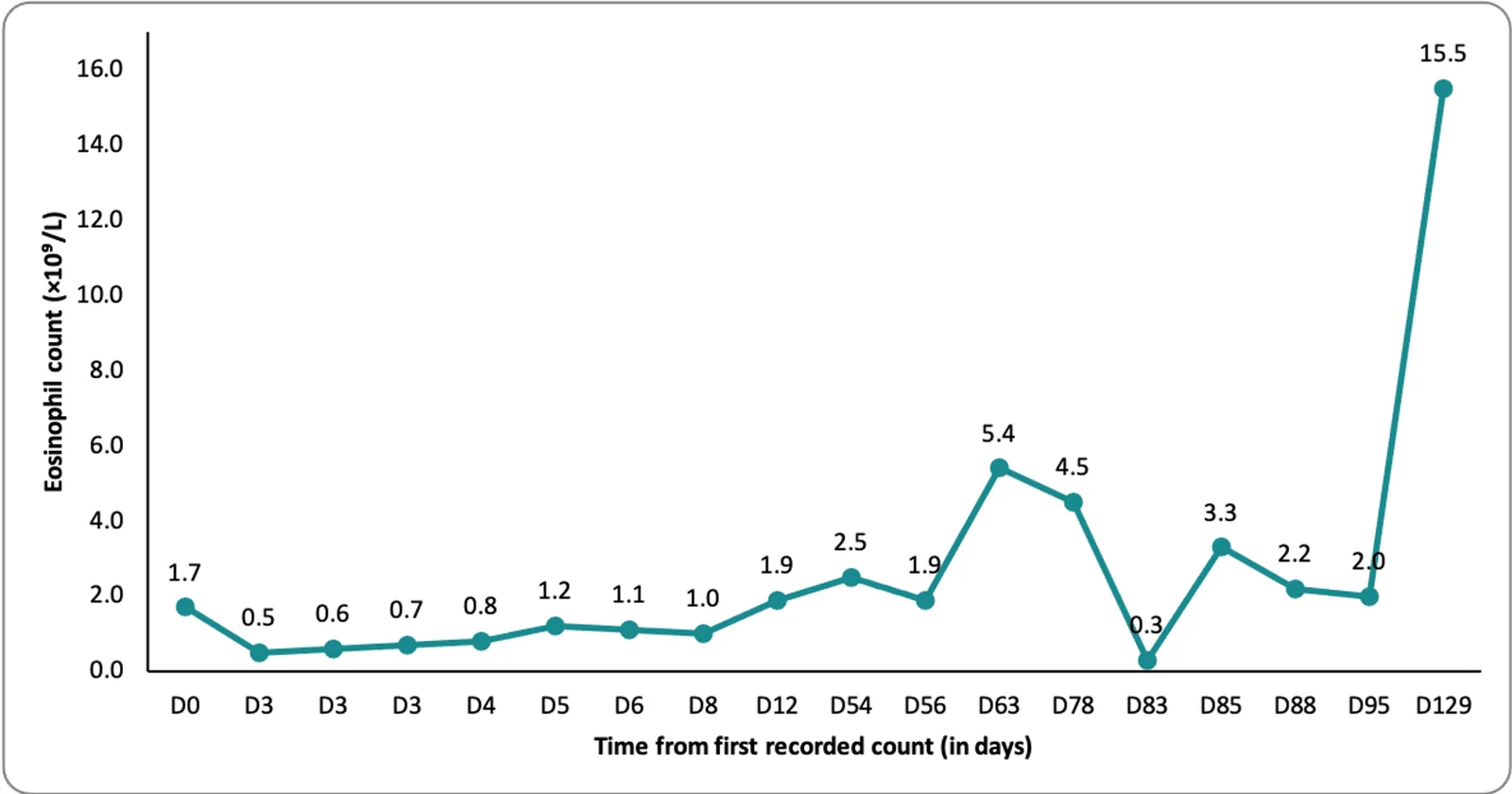
Serial peripheral blood eosinophil counts during the 18 weeks preceding diagnosis of eosinophilic granulomatosis with polyangiitis. Serial peripheral blood eosinophil counts recorded during multiple hospital admissions over the 18 weeks preceding diagnosis, demonstrating persistent eosinophilia with a progressive rise culminating in marked hypereosinophilia. The final measurement of 15.5 × 10⁹/L was obtained approximately seven weeks before the index presentation.

**Figure 2:**
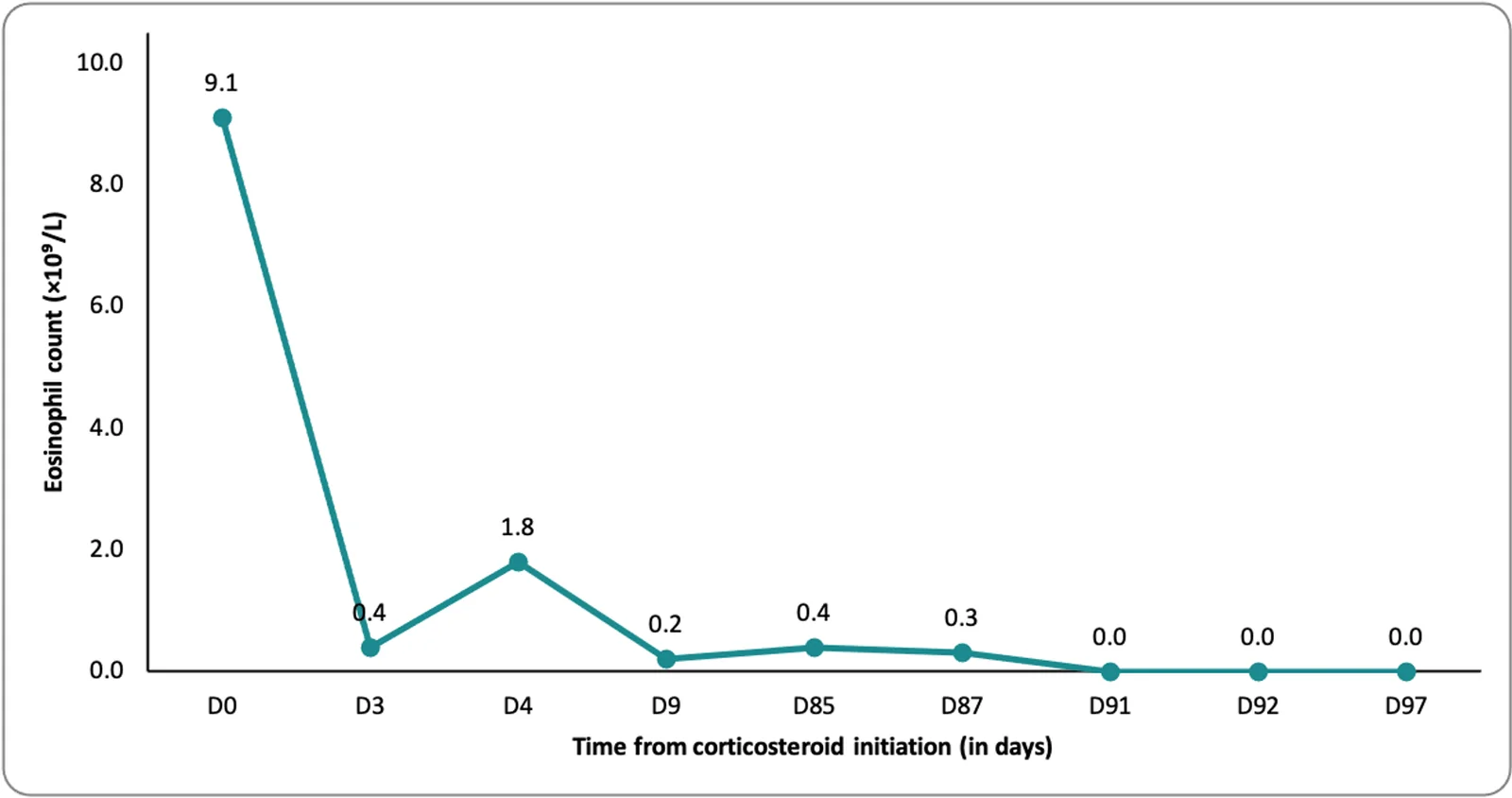
Peripheral blood eosinophil count on admission and after initiation of corticosteroid therapy. Day 0 represents the index hospital presentation, when the peripheral blood eosinophil count was 9.1 × 10⁹/L. Corticosteroid therapy was commenced on Day 1, with the eosinophil count decreasing to 0.4 × 10⁹/L by Day 3, approximately 48 hours after treatment initiation, demonstrating a rapid biochemical response.

Her regular medications included Trixeo® inhaler, salbutamol inhaler, Elleste Solo® 2 mg, Creon®, buprenorphine transdermal patch, oxycodone and pregabalin.

On presentation, her blood pressure was 160/88 mmHg, heart rate 108 beats/minute, respiratory rate 20 breaths/minute, oxygen saturation was 96% on room air, and temperature was 37.1°C. 

Clinical examination demonstrated palpable purpuric lesions affecting both lower limbs, feet, upper limbs and chest, with associated non-pitting oedema of the ankles. Multiple painful oral ulcers and nasal crusting were present (Figure 3). Respiratory examination demonstrated equal air entry bilaterally without focal wheeze or crepitations. Cardiovascular and abdominal examinations were otherwise unremarkable. There were no clinical features of neurological involvement, and electrocardiography demonstrated normal sinus rhythm.

**Figure 3:**
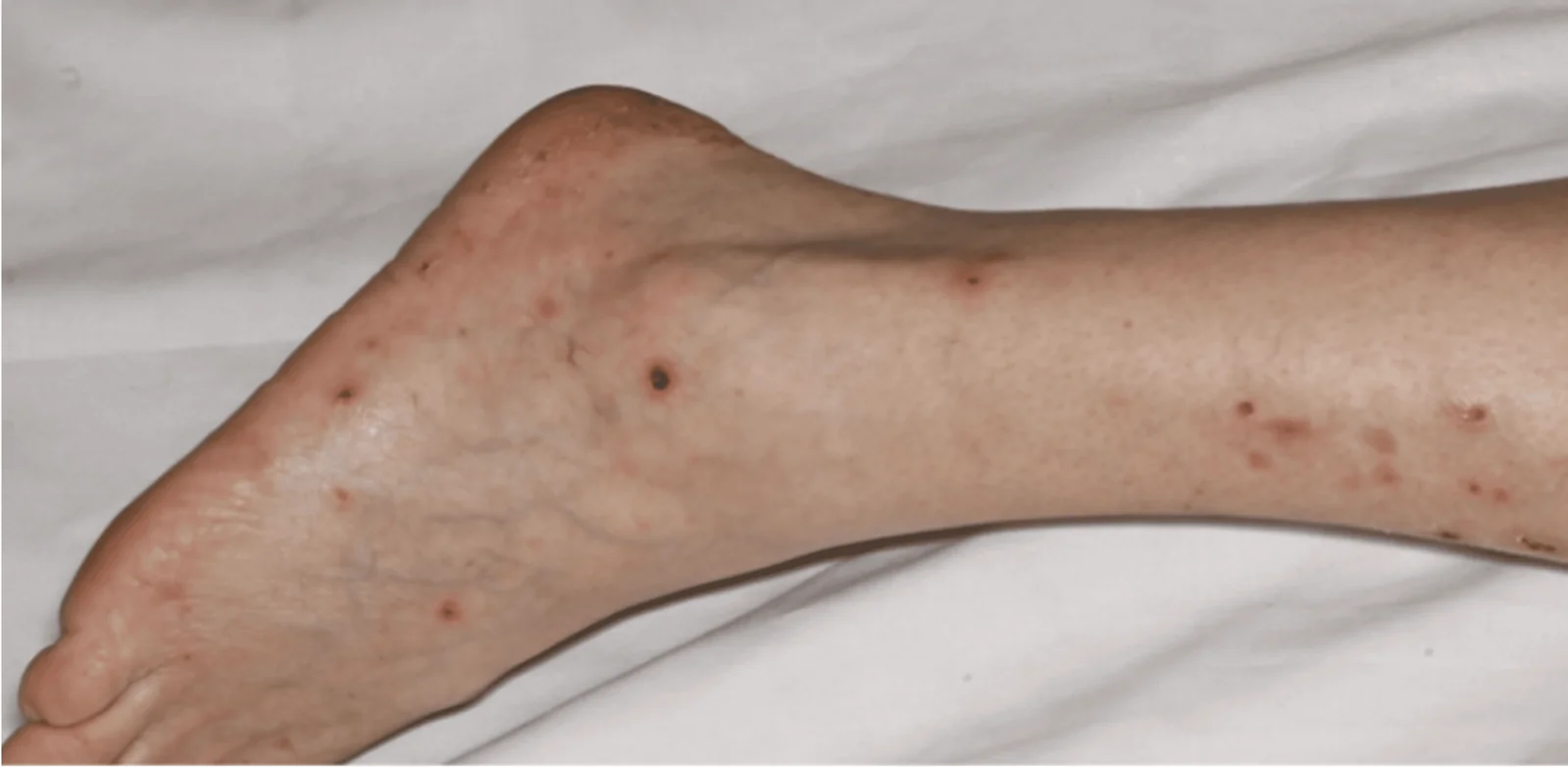
Clinical photograph demonstrating lower limb palpable purpura consistent with vasculitic skin involvement. Clinical photograph demonstrating multiple well-demarcated, non-blanching purpuric lesions affecting the right lower leg, clinically consistent with palpable purpura. Similar lesions were present on both lower limbs.

Initial laboratory investigations demonstrated a haemoglobin concentration of 132 g/L, white cell count of 17.5 × 10⁹/L and marked peripheral eosinophilia of 9.1 × 10⁹/L. C-reactive protein was elevated at 49 mg/L and erythrocyte sedimentation rate at 32 mm/hour. Renal function remained stable with a creatinine concentration of 93 µmol/L. Autoimmune serology demonstrated persistent low-positive PR3-ANCA (4.7 U/mL), while MPO-ANCA was negative (<0.2 U/mL). Antinuclear antibody, anti-double-stranded DNA antibody and anti-cyclic citrullinated peptide antibody were negative. Complement concentrations were mildly elevated (C3 2.44 g/L and C4 0.55 g/L) (Table 1).

**Table 1:** Summary of laboratory investigations at presentation. PR3-ANCA: proteinase 3 anti-neutrophil cytoplasmic antibody; MPO-ANCA: myeloperoxidase anti-neutrophil cytoplasmic antibody; ANA: antinuclear antibody; anti-dsDNA: anti-double-stranded DNA antibody; anti-CCP: anti-cyclic citrullinated peptide antibody; C3: complement component 3; C4: complement component 4 Reference ranges are those used by our institution.

Investigation	Result	Reference Range	Interpretation
Haemoglobin (g/L)	132	115-165	Normal
White Cell Count (x10⁹/L)	17.5	4.0-11.0	Elevated
Eosinophils (x10⁹/L)	9.1	0.0-0.5	Markedly elevated
C-reactive Protein (mg/L)	49	<5	Elevated
Erythrocyte Sedimentation Rate (mm/hour)	32	0-20	Elevated
Creatinine (µmol/L)	93	45-90	Stable renal function
PR3-ANCA (U/mL)	4.7	0.0-0.9	Low-positive result
MPO-ANCA (U/mL)	<0.2	0.0-0.9	Negative
ANA	Negative	Negative	Negative
Anti-dsDNA	Negative	Negative	Negative
Anti-CCP	Negative	Negative	Negative
Complement C3 (g/L)	2.44	0.90-1.80	Elevated
Complement C4 (g/L)	0.55	0.10-0.40	Elevated

Given the presenting haemoptysis and elevated D-dimer, computed tomography pulmonary angiography (CTPA) was performed. Pulmonary embolism was excluded; however, imaging demonstrated bilateral lower lobe bronchial wall thickening with mucus plugging, patchy ground-glass opacification and peribronchial inflammatory change consistent with inflammatory pneumonitis (Figure 4). A previously identified right upper lobe pleural-based pulmonary nodule remained stable. 

**Figure 4:**
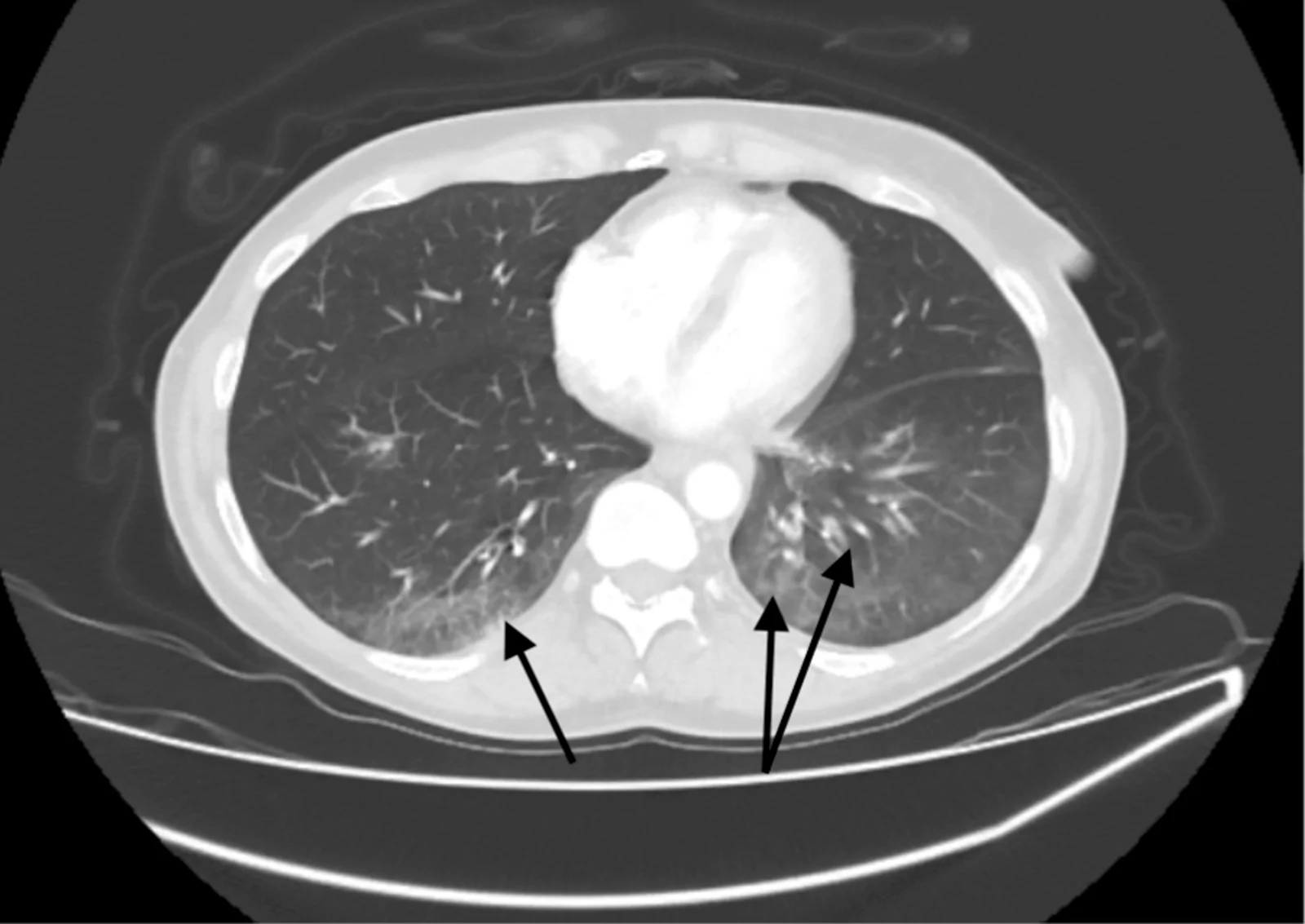
Computed tomography pulmonary angiography demonstrating bilateral lower lobe bronchial wall thickening with mucus plugging, patchy ground-glass opacification (arrows) and peribronchial inflammatory change consistent with inflammatory pneumonitis.

The patient was initially treated for presumed lower respiratory tract infection and bilateral lower limb cellulitis while further investigations were undertaken. Following multidisciplinary review by the rheumatology and respiratory teams, the clinical findings were considered sufficient to support a diagnosis of EGPA. The evolving multisystem clinical picture, together with the laboratory and radiological findings, was considered sufficient to initiate treatment without requiring histological confirmation. Tissue biopsy was therefore not pursued. High-dose oral prednisolone (60 mg once daily) was commenced together with proton pump inhibitor prophylaxis.

Following initiation of corticosteroid therapy, the patient's eosinophil count rapidly normalised to 0.4 × 10⁹/L within approximately 48 hours (Figure 2). Her cutaneous manifestations improved significantly, respiratory symptoms gradually resolved, and inflammatory markers declined. She was discharged on a tapering regimen of oral prednisolone with outpatient rheumatology follow-up. Approximately one month after discharge, she was readmitted with pulmonary haemorrhage attributed to EGPA. She was treated with intravenous methylprednisolone followed by a tapering regimen of oral prednisolone, with good clinical recovery during the admission.

## Discussion

This case demonstrates the diagnostic challenges associated with EGPA and highlights how the disease may evolve over a prolonged period before sufficient clinical features accumulate to support a diagnosis. During the two years preceding diagnosis, our patient required 10 hospital admissions and underwent three rheumatology assessments before developing the combination of eosinophilia, pulmonary inflammatory infiltrates, palpable purpura and upper airway involvement that ultimately resulted in a clinical diagnosis of EGPA. Importantly, this should not be viewed as a missed diagnosis but rather as an example of the progressive nature of EGPA and the clinical reality that the disease often only becomes recognisable as the characteristic clinical features emerge. A recent UK retrospective cohort study reported a median interval of 44 months between the first major symptom attributable to EGPA and formal diagnosis, with almost 87% of patients requiring review by at least one specialist during the year preceding diagnosis [6]. Although our patient received a diagnosis after approximately two years, her clinical course closely mirrors this recognised pattern of repeated specialist review and progressive disease evolution.

Earlier admissions were characterised by recurrent respiratory illness, chronic abdominal pain and intermittent cutaneous manifestations, while serial laboratory investigations demonstrated persistent eosinophilia with progressively increasing eosinophil counts. Given her pre-existing chronic pancreatitis, the relationship between her previous gastrointestinal symptoms and EGPA remains uncertain. Only later, did marked eosinophilia occur alongside pulmonary infiltrates, palpable purpura and upper airway disease, resulting in a more characteristic multisystem phenotype. The progressive eosinophil trend observed in this case highlights the importance of reviewing serial laboratory results rather than relying solely on findings from a single presentation. 

Marked peripheral eosinophilia is one of the defining laboratory features of EGPA and represents an important discriminator from other ANCA-associated vasculitides, including granulomatosis with polyangiitis and microscopic polyangiitis, where significant eosinophilia is uncommon. Although eosinophilia may occur in a wide range of conditions, persistent eosinophilia in combination with respiratory symptoms and extrapulmonary manifestations should prompt consideration of EGPA. In our patient, persistent low-positive ANCA serology alone was insufficient to establish the diagnosis. Instead, it was the presence of marked eosinophilia together with pulmonary, cutaneous and upper airway manifestations that represented the turning point in the diagnostic process.

Another notable feature of this case was the absence of a documented diagnosis of asthma despite established COPD and an active smoking history. Asthma is considered one of the hallmark features of EGPA and is present in most patients (reported in approximately 81% of a recent UK cohort), often preceding systemic disease by several years [3]. Consequently, our patient's recurrent respiratory symptoms were repeatedly attributed to community-acquired pneumonia and exacerbations of COPD, both of which represented reasonable alternative explanations at the time. Similar cases of delayed diagnosis in patients without classical asthma have been reported [7,8]. The absence of a documented asthma diagnosis may therefore have lowered clinical suspicion for EGPA during earlier admissions. However, historical spirometry, bronchodilator reversibility testing and fractional exhaled nitric oxide (FeNO) measurements were not available for review. Undiagnosed asthma or asthma-COPD overlap therefore cannot be excluded and may have contributed to some of her earlier respiratory presentations.

Several alternative diagnoses complicated the patient's clinical course. Recurrent respiratory infection and COPD accounted for many of her earlier pulmonary presentations, while persistent PR3-ANCA positivity prompted consideration of granulomatosis with polyangiitis and other ANCA-associated vasculitides. Her history of previous intranasal cocaine use warranted particular attention. Cocaine itself can cause destructive midline sinonasal lesions and ANCA positivity, often PR3-directed, producing a clinical and serological picture that can closely mimic ANCA-associated vasculitis [9]. Previous cocaine exposure therefore offered a plausible explanation for both the sinonasal destruction and persistent PR3-ANCA positivity and represented an important confounder in interpreting the serology. Ultimately, it was the emergence of multisystem involvement, with features not attributable to cocaine, that shifted the clinical picture towards EGPA. Although PR3-ANCA is itself uncommon in EGPA and is more frequently associated with granulomatosis with polyangiitis, it should be interpreted within the wider clinical context and pattern of disease [10,11].

Although the 2022 American College of Rheumatology/European Alliance of Associations for Rheumatology (ACR/EULAR) classification criteria have improved disease classification, they are not intended to establish a clinical diagnosis [11]. Our patient scored 5 points, based on peripheral eosinophilia ≥1 × 10⁹/L (+5), obstructive airway disease (+3) and PR3-ANCA positivity (-3), which is below the ≥6-point threshold for classification as EGPA [10]. Notably, PR3-ANCA positivity is negatively weighted within these criteria and therefore did not support classification as EGPA. Clinical judgement remained central to the diagnosis in this case, which was made following specialist multidisciplinary assessment once sufficient multisystem clinical features had emerged. 

Current treatment recommendations advocate systemic corticosteroids as first-line therapy for remission induction, with additional immunosuppressive or biologic agents reserved for patients with severe disease, organ-threatening manifestations or relapsing disease [12]. Our patient demonstrated a rapid clinical and biochemical response following initiation of high-dose corticosteroid therapy, with normalisation of eosinophil counts within approximately 48 hours and marked improvement in respiratory and cutaneous symptoms. In retrospect, the patchy ground-glass opacification noted at presentation may have represented early alveolar haemorrhage, given her subsequent readmission with overt pulmonary haemorrhage, underscoring the potential for rapid progression to organ-threatening disease.

Several published case reports have similarly described prolonged diagnostic intervals resulting from the heterogeneous and evolving presentation of EGPA [7]. However, our case is distinguished by repeated hospital admissions over a prolonged period, multiple rheumatology assessments and the coexistence of several plausible alternative diagnoses, including COPD, recurrent respiratory infection and cocaine-associated destructive sinonasal disease. Together, these factors delayed recognition until the characteristic phenotype became fully established. The serial eosinophil trend included in this report further illustrates how disease evolution may be appreciated over time. 

This case highlights several important learning points for clinical practice. EGPA should remain an important differential diagnosis in patients with persistent eosinophilia and recurrent multisystem manifestations, even when more common diagnoses appear to explain individual presentations. Serial clinical assessment and review of previous investigations may reveal an evolving pattern that is not apparent during isolated presentations. Early recognition of this evolving pattern of disease may facilitate timely diagnosis and initiation of immunosuppressive therapy.

We acknowledge that this case has several limitations. The diagnosis of EGPA was made clinically without histopathological confirmation, as the treating multidisciplinary team considered the evolving multisystem presentation sufficient to support the diagnosis and initiate treatment. A comprehensive investigation specifically excluding all alternative causes of hypereosinophilia was not documented; therefore, competing eosinophilic disorders cannot be formally excluded retrospectively. Historical spirometry, bronchodilator reversibility testing and FeNO measurements were also unavailable, limiting retrospective assessment for undiagnosed asthma or asthma-COPD overlap. These limitations should be considered when interpreting the case.

## Conclusions

This case demonstrates the diagnostic challenges of EGPA and how the disease may evolve over years before sufficient clinical features emerge to support a clinical diagnosis. Recurrent non-specific presentations, when accompanied by persistent eosinophilia and progressive multisystem manifestations, should prompt ongoing clinical reassessment, even when earlier investigations are inconclusive. Recognition of changing clinical features can facilitate earlier diagnosis and timely initiation of appropriate treatment.

## References

[1] Fijolek J, Radzikowska E (2023). Eosinophilic granulomatosis with polyangiitis - advances in pathogenesis, diagnosis, and treatment. Front Med (Lausanne).

[2] Chakraborty RK, Rout P (2025). Eosinophilic granulomatosis with polyangiitis (Churg-Strauss syndrome). StatPearls [Internet].

[3] Hwee J, Harper L, Fu Q, Nirantharakumar K, Mu G, Jakes RW (2024). Prevalence, incidence and healthcare burden of eosinophilic granulomatosis with polyangiitis in the UK. ERJ Open Res.

[4] Dolin P, Lucas S, Gamble A, Turner M, Rowell J (2025). Systematic literature review and meta-analysis of the epidemiology and clinical burden of eosinophilic granulomatosis with polyangiitis. Mod Rheumatol.

[5] Dereseviciene G, Dadoniene J, Miltiniene D (2025). Similarities and differences between patients diagnosed with ANCA-associated vasculitis who are positive and negative for ANCA: university clinic practice and expertise. Medicina (Kaunas).

[6] Siddiqui S, Dolin P, Shavit A (2024). The diagnostic journey of patients with eosinophilic granulomatosis with polyangiitis (EGPA) in England: a retrospective observational cohort study. Thorax.

[7] Alam V, Nanzer AM (2022). Eosinophilic granulomatosis with polyangiitis: case report and literature review. Breathe (Sheff).

[8] Hagemann J, Laudien M, Becker S (2024). EGPA: eosinophilic granulomatosis with polyangiitis (Churg-Strauss syndrome) as a special presentation of chronic rhinosinusitis with nasal polyps (CRSwNP). Allergol Select.

[9] Trimarchi M, Gregorini G, Facchetti F (2001). Cocaine-induced midline destructive lesions: clinical, radiographic, histopathologic, and serologic features and their differentiation from Wegener granulomatosis. Medicine (Baltimore).

[10] Papo M, Sinico RA, Teixeira V (2021). Significance of PR3-ANCA positivity in eosinophilic granulomatosis with polyangiitis (Churg-Strauss). Rheumatology (Oxford).

[11] Grayson PC, Ponte C, Suppiah R (2022). College of Rheumatology/European Alliance of Associations for Rheumatology classification criteria for eosinophilic granulomatosis with polyangiitis. Ann Rheum Dis.

[12] Biddle K, Jade J, Wilson-Morkeh H (2025). The 2025 British Society for Rheumatology management recommendations for ANCA-associated vasculitis. Rheumatology (Oxford).

